# Contraceptive use intentions and unmet need for family planning among reproductive-aged women in the Upper East Region of Ghana

**DOI:** 10.1186/s12978-019-0693-x

**Published:** 2019-03-04

**Authors:** Ayaga A. Bawah, Patrick Asuming, Sebastian F. Achana, Edmund W. Kanmiki, John Koku Awoonor-Williams, James F. Phillips

**Affiliations:** 1 Regional Institute for Population Studies, University of Ghana, Accra, Ghana; 2 University of Ghana Business School, University of Ghana, Accra, Ghana; 3 Navrongo Health Service Research, Ghana Health Service, Navrongo, Ghana; 4 Ghana Health Service, Headquarters, Accra, Ghana; 5 Mailman School of Public Health, Columbia University, New York, USA

**Keywords:** Contraception, Unmet need, birth spacing, Family planning, Reproductive health, Fertility

## Abstract

**Background:**

Motivations for use of contraceptives vary across populations. While some women use contraceptives for birth spacing, others adopt contraception for stopping childbearing. As part of efforts to guide the policy framework to promote contraceptive utilization among women in Ghana, this paper examines the intentions for contraceptive use among reproductive-aged women in one of the most impoverished regions of Ghana.

**Methods:**

This paper utilizes data collected in 2011 from seven districts in the Upper East Region of northern Ghana to examine whether women who reported the use of contraceptives did so for the purposes of stopping or spacing childbirth. A total of 5511 women were interviewed on various health and reproductive health related issues, including fertility and family planning behavior. Women were asked if they would like to have any more children (for those who already had children or those who were pregnant at the time of the survey).

**Results:**

The prevalence of contraceptive use was low at 13%, while unmet need is highly pervasive and demand for family planning is predominantly for spacing future childbearing rather than for the purpose of stopping. Overall, about 31.7%of women not using contraceptives reported a need for spacing while 17.6% expressed a need for limiting. Thus, the latent demand for family planning is dominated by preferences for space rather than limiting childbearing.

**Conclusion:**

Results show that there is latent demand for family planning and therefore if family planning programs are appropriately implemented they can yield the desired impact.

## Plain English summary

Motivations for using contraceptives are not the same across different populations. Some women use contraceptives for spacing childbirth while others use them for purposes of stopping childbirth. As part of efforts to guide the policy framework to promote contraceptive utilization among women in Ghana, this paper examines the intentions for contraceptive use among reproductive-aged women in one of the most impoverished regions of Ghana. Using data collected in 2011 from seven districts in the Upper East Region of northern Ghana, the paper assesses whether women who reported using contraceptives did so for the purposes of stopping or spacing childbirth. And, for those who did not use we examined whether they have an unmet need i.e. expressed a desire for spacing or stopping childbearing even though they were not using any method of contraception that will help them to realize those desires.

A total of 5511 women were interviewed on various health and reproductive health related issues, including fertility and family planning behavior. Women were asked if they would like to have any more children (for those who already had children or those who were pregnant at the time of the survey). The contraceptive usage was found to be low at 13%, although there was a high need for contraceptive use. Most women required family planning for spacing future childbearing rather than for the purpose of stopping childbirth. Overall, about 31.7% of women not using contraceptives reported a need for spacing while 17.6% expressed a need for limiting birth. Therefore, majority of women who require family planning need it for spacing birth rather than reducing the number of children.

Results show that there is a high need for family planning and therefore if family planning programs are appropriately implemented they can yield the desired impact.

## Introduction

Contraceptive use interventions and unmet need for family planning are important determinants of fertility decline in both developed and developing countries. [1].It is often argued that for sustained fertility decline to occur, there must be a deliberate effort on the part of women to limit childbearing (parity-specific deliberate control) [1]. Women may choose to limit fertility by using contraceptive methods to either space or stop childbearing.

There is ample evidence from analyses of Demographic and Health Surveys (DHS) and other data in sub-Saharan Africa that show that contraceptive use is related to both child spacing and stopping behavior [2–4]. On the other hand, there is substantial evidence to show that while many women in sub-Saharan Africa sometimes report that they do not want to have any children at all or probably did not want to have them at a particular time that they did, they often do not use modern contraceptives methods to ensure that they achieve those desires. Such women are reported to have an unmet need for family planning [5–7]. The number of women with this discrepant behavior continues to be significant despite family planning and other fertility regulation interventions introduced in many low and middle-income countries to give women opportunities for regulating childbearing [6]. The reason for this can be attributed to several factors, including lack of knowledge of family planning methods, limited availability of family planning services or concerns about moral and social acceptability [8, 9].

This paper examines contraceptive use or non-use intentions and unmet need for family planning and tries to understand the motivations for use or none use of contraceptives, by women who express desire for either spacing or stopping childbearing. It is important to examine both the motivations for use or non-use of contraceptives (particularly by women who express a desire to either space or stop childbearing) because it will shed light on how programme managers should craft interventions to make the desired impact. The reason why we chose to examine both the motivations by those using contraceptives and those not using even though they have a need for contraception (unmet need) is because the two groups of women may seem to either mirror image each other or may have contrasting motivations, which when taken together could give a comprehensive picture of the issues. The analysis is based on data from one of the poorest regions of Ghana where contraceptive use is low. Results are intended to clarify the extent to which unmet need for contraception and use of contraception in such rural environments may be related to spacing and limiting fertility behavior.

### Context of study setting

The paper is based on data from the Upper East region (UER) of Ghana where a plausibility trial known as the Ghana Essential Health Intervention Programme (GEHIP) was implemented. GEHIP was implemented to address concerns that Ghana’s community health delivery system known as Community-based Health Planning & Services (CHPS) originally piloted and successfully implemented in the Kasena-Nankana District of UER was losing traction. GEHIP was therefore a packaged of coordinated interrelated health systems interventions aimed at improving maternal and child mortality [11]. At the time of its implementation UER was one of the poorest regions in the country [12]. The region ranked among the poorest 5% of Ghana’s districts in 2009 when the programme was starting, with the economies of districts dominated by subsistence agriculture. According to the Ghana Statistical Service (GSS), per capita income for these districts was about a quarter that of Ghana, ranking equivalently with the districts of the Upper West Region as the two most impoverished regions of Ghana [11].

Against this backdrop of profound economic adversity, the region is also health service deprived. With one regional hospital located in the regional capital that served as a referral facility for the over 10 districts, the hospital itself lacked basic specialized care, apart from obstetrical care. However, while tertiary health care is poorly developed, community-based primary health care has become more accessible in recent years, providing access to basic curative and preventive health services for children. The GEHIP interventions were therefore aimed at addressing these health adversities.

To help evaluate the impact of GEHIP, a baseline survey was conducted in 2011 in seven rural districts of Upper East Region where interventions of the GEHIP project were implemented. The questionnaire that was used to collect the data for the evaluation was adapted from the Ghana Demographic and Survey data collection instruments with adaptation to capture the local context. This paper utilizes the data from the baseline survey to measure contraceptive rates, intentions of women for using contraceptives and to measure unmet need for family planning in this rural part of Ghana.

### Theoretical background and motivations

Coale’s principle of calculus of conscious choice posits that for fertility to decline, there must be conscious efforts on the part of women to reduce the number of children they will want to give birth to, facilitated by the availability of fertility regulation mechanisms [10]. This is perhaps one of the key guiding principles that led to theoretical conceptualizations of reproductive intentions and desires anchored on principles of conscious choice. The idea is that the decision to have children, when to have them and how many to have, is based on calculations of women (and in some cases with their partners), depending on various considerations that they translate into fertility decisions. This has led demographers to design questions that are fielded in surveys aimed to capture the intentions of women regarding their fertility that might help in their design of programs [11]. Therefore, women are often asked questions as to whether they would want to have children, how many and when? These questions are asked of all women who report that they are fecund.

## Methods

### Study setting

**Fig. 1:**
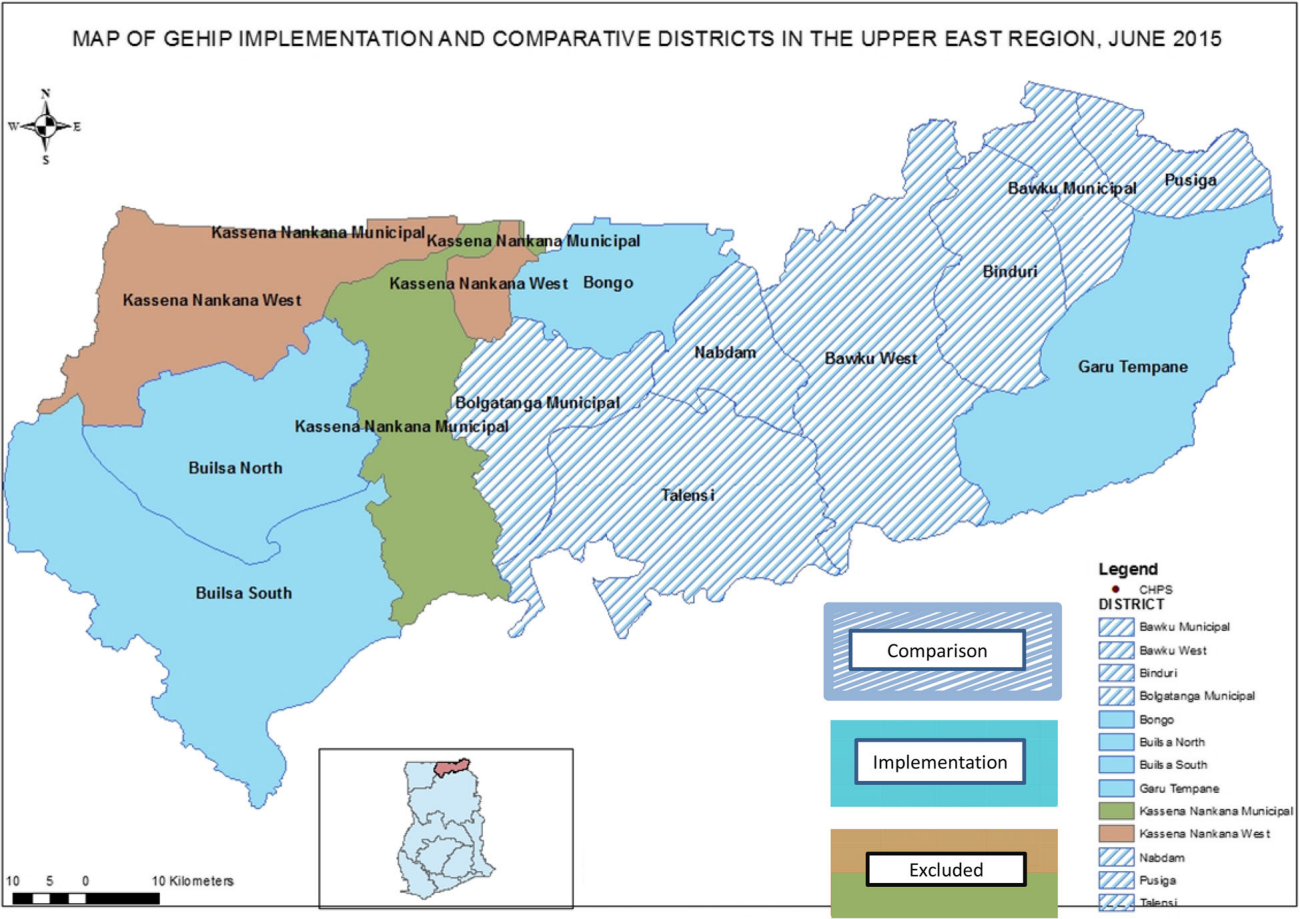
Map of the GEHIP Implementing and Comparison Districts in the Upper East Region (Insert shows Upper East Region relative to Ghana)

Data for this study were collected in the Upper East Region (UER) of northern Ghana. This region is located in the north-eastern corner of Ghana and bordered by Burkina Faso to the north and Togo to the east, to the west by the Sissala District in the Upper West Region and to the south by the West-Mamprusi District of the Northern region of Ghana. The region lies between longitude 0° and 1° west, and latitudes 10° 30′N and 11°N. The climate is characterized by one erratic rainy season from May/June to September/October and a long spell of dry season spanning November to mid-February, characterized by cold, dry and dusty harmattan winds (Fig. 1).

The Upper East Region is made up of 13 administrative districts but at the time of the survey, there were only 9 districts. However, 4 additional districts were later created by the central government through the splitting of existing districts making a total of 13 districts now. The survey was conducted in seven of the then 9 districts.

The region inhabits 4.2% of Ghana’s estimated 28 million people, predominantly rural (79%) and is one of Ghana’s poorest regions. [12].The economy is primarily agrarian, poverty is pervasive, while fertility and mortality levels are relatively high and demand for fertility regulation is relatively low [11, 12].

Illiteracy is quite pronounced in the region, with as high as 44.5% of the people reported as never been to school. Early marriage and childbearing and their associated adverse consequences are common. The total fertility rate (TFR) in the region is 4.1 compared to national TFR of 2.5 in Greater Accra; the national capital [13].

### Data collection

This paper draws on data collected through a randomized cluster survey conducted in the Upper East Region. The survey is part of a health systems intervention project called the Ghana Essential Health Intervention Program (GEHIP). Details of GEHIP can be found elsewhere [14, 15]. A total of 5511 women were interviewed on various health and reproductive health related issues, including fertility and family planning behaviors. Based on a two staged sampling procedure, a total of 6000 women of reproductive age (15–49) were drawn to be interviewed. The first stage involved a random sampling of selected Enumeration Areas (EAs) using a universe of EAs provided by the Ghana Statistical Service (GSS). The EAs covered both the intervention and non-intervention districts. A total of 66 EAs were selected.

All women in these EAs were subsequently listed and then a random sample of 6000 women were then selected at the second stage for interviewing. The number of women interviewed per EA is based on the population of the EA. Sample proportions based on the relative population size of each of the 66 EAs were calculated which were used as weights to determine the number of women to interview in each EA. Households of the selected women were visited and the said women identified and interviewed. Of the total 6000 women targeted, 5511 women were successfully interviewed representing a success rate of 91.8%. Among many other things, information on the households to which the women were drawn was collected, background characteristics of the women, their reproductive histories, contraceptive use and reasons for use or non-use, fertility intentions, etc. In order to situate the estimates obtained from the analysis of the GEHIP data we compared estimates of TFR, Contraceptive use and unmet as obtained from our data with those reported by the 2008 Ghana Demographic and Health Survey and also from the Population Reference Bureau [16, 17].

### Consent to participate

During the survey, two levels of informed consent was obtained (household level and individual woman level). Enumerators first approach heads of sampled households, explain the purpose of the study, requirements for participation, risk and benefits among others and seek their permission to interview respondents within their household. Upon approval from the household head, Enumerators then approach individual women within selected households and also seek their consent to participate in the survey through the same process. For those women who consent to participate, two copies of the consent form are endorsed by the enumerator and the respondent and one copy given to the respondent before the interview takes place.

### Data analysis

In addition to the tabulation of bivariate relationships, a multinomial logit model is used in examining the determinants of respondent classification into a five-category polytomous outcome variable (non-users who want to space childbearing; non-users who want to limit childbearing; users whose reason for use is spacing; users whose reason for use is limiting childbearing, and non-users who want more children or have no need for contraception) on the implementation of reproductive preferences conditional on various independent variables. The first group of women (non-users who either want to space or limit) are those who have either unmet need for spacing or limiting; the second group are those whose needs have been met (i.e. those who are using because they want to space or limit child birth), and finally the last group of women are those who are not using because they want to have children. The model is of the form:

1 $$ \Pr \left({y}_i=j\right)=\frac{\exp \left({x}_i{\beta}_j\right)}{\sum_j^J\exp \left({x}_i{\beta}_j\right)} $$

where pr(y_i_ = j) is the probability of belonging to group j, x_i_ is a vector of explanatory variables including educational attainment, age, parity, marriage type, women’s work status, religion and characteristics of spouses as independent variables, and ***β*** are corresponding coefficients (reported in terms of relative risks), which are estimated using maximum likelihood estimation [18].

We excluded respondents who are not currently in a marital union because we were only interested in women who are married. The frequency distribution of the “j” classes of the dependent variable are presented in the bottom row of Table 2 as i) non-users who have no need for family planning, ii) those using to limit childbearing, iii) fecund, not using, and expressing a need to space future childbearing, (iv) fecund, not using, and expressing a preference to limit childbearing, and v) an omitted class comprised of women are using contraceptives to space childbearing.

## Results

Table 1 reports results of the total fertility rate, contraceptive prevalence rate and unmet need for spacing and limiting from the GEHIP survey for the Upper East region, comparing it to estimates from the Ghana Demographic and Health Survey (GDHS) and other sources, for the region. The results from the GEHIP survey portray low levels of contraceptive use, high levels of fertility and unmet need for both spacing and limiting for the region, when compared with the GDHS results.

**Table 1:** Fertility, contraceptive prevalence, and unmet need for family planning among women aged 15–49, Africa, Ghana, Upper East Region, and GEHIP Districts

Indicator	Sub-Saharan Africa^a^	Ghana		
		National^b^	All Upper East^b^	GEHIP Baseline Survey^c^
Total fertility rate	5.2	4.0	4.1	5.4
Prevalence of modern method contraceptive users among currently married women aged 15–49	23	16.6	14.3	12.9
Prevalence of all contraceptive users among women aged 15–49	19	23.3	14.7	14.1
Unmet need for spacing^d^	17	22.5^b^	22.7	31.7
Unmet need for limiting^d^	9	12.9^b^	9.4	17.6

^a^Population Reference Bureau, 2011

^b^Based on sample census enumeration clusters in all nine districts, including urban Bolgatanga and district headquarter towns (Demographic and Health Survey, Ghana Statistical Service, GDHS, 2008)

^c^Based on rural census enumeration areas only and excluding two research districts of the Navrongo Health Research Centre, Kassena-Nankana East and West.

^d^Among currently married women

For instance, the TFR obtained from the GEHIP survey was 5.4 compared to a TFR of 4.0 reported in the 2008 GDHS, while the contraceptive prevalence rate for modern methods was 13.0%, compared with 14.3% from the 2008 GDHS. These results suggest that there is a prominent potential for the program to have an effect, owing to the high prevalence of unmet need for family planning among currently married women. About 31.6% of all women who were interviewed indicated that they wanted to space and yet reported that they were not using a method of family planning at the time of the survey. A far lower proportion wanted to limit childbearing yet were not using any method at the time of the survey. In all, 17.6% of the currently married women reported that they no longer wanted to have children but were nevertheless not using any method of family planning.

### Reproductive preferences by background characteristics

Cross-tabulations presented in Table 2 portray differentials in met and unmet need. Among all the non-users, majority of them expressed a preference for spacing rather than limiting. For instance, 51.8% of women below the age of 30 who were not using any method of family planning at the time expressed a desire to space compared to only 2.5% of those in similar age bracket who preferred limiting. Similarly, of those whose needs were met, many of them were using contraceptives for spacing purposes (19.7% for spacing versus 0.77% for limiting). However, as women aged, a higher proportion (users and nonusers) prefer contraception for purposes of stopping rather than spacing. For example, only 2.5% of the women below age 30 who were not using expressed a preference for stopping compared to 48.7% among those who are 40 years and above. A similar situation obtains among those who are using. As women aged, a greater proportion of them use contraceptives for purposes of stopping. Similarly, many higher parity women who are not using a method of contraception expressed a desire to stop (38.5% among women with 5 or more children, compared to 6.1% for those with 1–4 children and 0.8% for those without children at all).

**Table 2:** Background characteristics of currently married women aged 15–49 by categories of reproductive preferences and behavior, GEHIP Baseline Survey, Upper East Region of Ghana

Covariates	Non-users who have no need for family planning		Contraceptive use status by preference class:								All Respondents in each class:	
			Unmet need: Non-users				Met need: contraceptive users					
			Wants to space future deliveries		Wants to limit additional fertility		Using to space future deliveries		Using to limit additional fertility			
	Cases	%	Cases	%	Cases	%	Cases	%	Cases	%	Cases	%
Age:												
< 30	295	25.11	609	51.83	30	2.55	232	19.74	9	0.77	1175	100.0
30–39	314	27.67	408	35.95	187	16.48	162	14.27	64	5.64	1135	100.0
40+	212	27.93	91	11.99	370	48.75	35	4.61	51	6.72	759	100.0
Parity:												
No child	93	72.66	29	22.66	1	0.78	5	3.91	0	0.00	128	100.0
1–4 children	410	24.36	806	47.89	102	6.06	345	20.50	20	1.19	1683	100.0
5 + children	318	25.28	273	21.70	484	38.47	79	6.28	104	8.27	1258	100.0
Educational attainment:												
None	647	27.76	812	34.83	506	21.71	270	11.58	96	4.12	2331	100.0
Some	173	23.47	296	40.16	81	10.99	159	21.57	28	3.80	737	100.0
Unknown											1	
Marriage type:												
No Union	16	30.19	17	32.08	11	20.75	7	13.21	2	3.77	53	100.0
Monogamous	454	24.29	731	39.11	291	15.57	318	17.01	75	4.01	1869	100.0
Polygynous	351	30.60	360	31.39	285	24.85	104	9.07	47	4.10	1147	100.0
Economic indicators:												
Employed	700	26.66	918	34.96	531	20.22	364	13.86	113	4.30	2626	100.0
Not employed	121	27.31	190	42.89	56	12.64	65	14.67	11	2.48	443	100.0
Own Cellphone	235	28.52	270	32.77	113	13.71	172	20.87	34	4.13	824	100.0
No Cellphone	586	26.10	838	37.33	474	21.11	257	11.45	90	4.01	2245	100.0
No Radio in HH	239	28.12	286	33.65	174	20.47	124	14.59	27	3.18	850	100.0
Listen to Radio	580	26.22	820	37.07	412	18.63	304	13.74	96	4.34	2212	100.0
Unknown	2	28.57	2	28.57	1	14.29	1	14.29	1	14.29	7	100.0
TV Viewing												
Views TV	239	27.19	306	34.81	142	16.15	145	16.50	47	5.35	879	100.0
No Viewing	580	26.56	801	36.68	444	20.33	284	13.00	75	3.43	2184	100.0
Unknown	2	33.33	1	16.67	1	16.67	0	0.00	2	33.33	6	100.0
Total currently married women	**821**	**26.75**	**1108**	**36.10**	**587**	**19.13**	**429**	**13.98**	**124**	**4.04**	**3069**	**100.0**

### Effect of women’s characteristics on reproductive preferences and behavior

Multinomial regression results presented in Table 3 show that the progression of age and parity are associated with increased realization of reproductive preferences. The reference category, as noted above, refers to women who are currently using contraceptive and indicated that their reason for use is to space childbirth. Relative to current users for spacing, women with an unmet need are more likely to have a desire for stopping purposes as shown by the significantly higher and positive relative risks ratios of 1.19. A similar pattern is observed for currently using women. As age increases, women who are currently using contraception are more likely to be using for purposes of stopping rather than spacing. Parity is also associated with limiting behavior. For instance, relative to women currently using contraceptives for spacing, women who are not using any method are more likely have a desire to stop child bearing as shown by the relative risk ratio of 1.97 which is statistically significant. Even among the users, as age increases use of contraception is more likely to be associated with limiting behavior as shown by the significantly higher risk ratio of 2.35. Exposure to television is also associated with limiting behavior. Women living in households that have television are more likely to use contraceptive for limiting compared to those who use for spacing. Somehow, educational attainment is less likely to be associated with unmet spacing behavior and unrelated to limiting.

**Table 3:** Multinomial logistic regression relative risk ratios statistical tests for the effect of background characteristics of women aged 15–49 on reproductive preferences and behavior, GEHIP Baseline Survey

Covariates:	Contraceptive use status by preference class compared with using to space							
	Non-Users		Unmet need for spacing		Unmet need for limiting		Using to limit additional fertility	
	RRR	Z	RRR	Z	RRR	Z	RRR	Z
Age	0.99	+ 0.43	0.94	−3.40**	1.19	+ 6.27***	1.15	+ 3.13**
Parity	0.39	−6.50***	0.79	−1.75	1.52	+ 1.97*	2.35	+ 2.84**
Age-parity interaction	1.03	+ 6.17***	1.01	+ 2.26*	1.00	+ 0.10	0.99	−0.98
Some educational attainment	0.58	−3.68***	0.67	−2.92**	0.95	−0.26	1.24	+ 0.79
Monogamous marriage	0.85	−0.34	1.02	+ 0.04	1.80	+ 1.06	2.14	+ 0.90
Polygyny marriage	1.60	+ 0.98	1.51	+ 0.88	2.62	+ 1.73	2.06	+ 0.85
Employed	0.73	−1.80	0.70	−2.14*	0.66	−1.84	0.93	−0.20
Household with 1 or more cellphones	0.71	−2.58*	0.55	−4.63***	0.65	−2.47*	0.90	−0.41
Household with 1 or more radios	1.02	+ 0.15	1.28	+ 1.87	1.00	−0.00	1.50	+ 1.59
Respondent views television	0.96	−0.32	0.87	−1.09	1.09	+ 0.54	1.86	+ 2.66**
Summary statistics:								
Number of observations:	3207							
Likelihood R^2^:	1350.83 (40 d.f.)							
*P* value:	0.000 ***							
Psuedo- R^2^:	0.1470							

* *p* < 0.05 ** *p* < 0.01 *** *p* < 0.001; *a) Omitted class = using for spacing; b) RRR = Relative risk ratio*

Table 4 presents results of an analysis of the sample of women who reported using contraceptives for spacing or limiting relative to non-users, with women who are using contraceptives for purposes of spacing as the reference category. Results demonstrate how the decision to adopt family planning is associated with preferences. Women who have achieve a relatively high parity for their age are far more likely to have adopted contraception to limit childbearing (1.49 times more likely for limiting as opposed to spacing). Also, as age increases women are more likely to use contraceptives to limit fertility.

**Table 4:** Multinomial logistic regression relative risk ratios statistical tests for the effect of background characteristics of women aged 15–49 on reproductive preferences among contraceptive users, GEHIP Baseline Survey

Covariates:^a^	Contraceptive use status compared with using to space			
	Non-Users of contraceptives		Using to limit additional fertility	
	RRR^b^	Z	RRR	z
Age	1.04	+ 3.92^***^	1.01	+ 2.71**
Parity	0.06	+ 1.59	1.49	+ 6.27***
Some educational attainment	0.71	−2.70**	1.12	+ 0.43
Monogamous marriage	1.01	+ 0.03	2.16	+ 0.92
Polygynous marriage	0.61	+ 1.11	2.17	+ 0.92
Employed with monetary income	0.68	+ 2.44*	1.02	+ 0.04
Household with 1 or more cellphones	0.58	−4.73***	0.80	−0.88
Household with 1 or more radios	1.18	−1.41	1.47	+ 1.52
Respondent views television	1.92	−0.74	1.81	+ 2.58*
Summary statistics:				
Number of observations:	3207			
Likelihood R^2^:	262.03 (18 d.f.)			
P value (d.f.):	0.000**			
Psuedo- R^2^:	0.0743			

^a^ *Omitted class = using for spacing; b) RRR = Relative risk ratio*

* p < 0.05 ** p < 0.01 *** *p* < 0.001

However, the results for the education are somewhat curious. There seems to be no difference in the relative likelihood that educated women will use modern contraception to limit or to space childbirth. However, educated women are less likely to be among the non-users or in the unmet need for spacing group, compared to being in the group who are using modern contraception to space. Women residing in households where there is a television set are also more likely to use for limiting.

In general, attributes of women such as parity, educational attainment and ownership of television are associated with the use of contraception to limit rather than space childbearing.

## Discussion

This study has examined contraceptive use intentions, unmet need for contraception and their associated determinants among married women in the Upper East Region of Ghana. First, the results presented seem to suggest that the national CHPS scale-up policy between the year 2000 and 2010 has not been associated with a transformation of reproductive behavior or a replication of similar results of the Navrongo Experiment [16]. This is because contraceptive use levels have remained relatively low in the UER Region shown by the results, as is found elsewhere in Ghana. Unmet need for family planning remains pervasive, with most of the demand for family planning found among non-users of contraception expressing intentions for spacing births. Compared with the levels of unmet need reported in the 2014 GDHS, our results show unusually higher levels of unmet need for both spacing and limiting. The higher estimates obtained from our analysis could be due to the fact that the GEHIP survey was restricted to the rural parts of the region and the exclusion of the two Kassena-Nankana districts where the Navrongo project was carried out and shown to have had significant increases in contraceptive use and fertility regulation.

Results suggest that older women who seek to limit fertility are more effective implementers of their reproductive preference than younger women, as illustrated by the significant relative risk ratio. More importantly, the net effect of parity, controlling for age, is associated with the desire to limit childbearing – a preference that contributes equivalently to “met” and “unmet need”. Unsurprisingly, women who have achieved high parity for their age tend to use contraception more for limiting purposes. Finally, somewhat interestingly, meeting the need for contraception either for spacing or limiting is associated with educational attainment, whereas educated women expressing unmet need indicated a need to space rather than limit. This is women with some education are almost equally likely to be using modern contraception to space or have an unmet need for limiting. This suggests that education seem to matter most irrespective of whether a woman is a non-user or has an unmet need for spacing on one hand, or such a woman is using contraception some other reason, on the other. It means there is both unfulfilled demand for contraception among educated women whether for spacing, limiting or some other reason.

These results imply a potentially high latent demand that if mobilized could increase contraceptive use in this impoverished region of northern Ghana. Results also suggest, however, that the promotion of family planning should address the need for convenient, safe, and effective methods of spacing in addition to exploring other mediums for introducing concepts of reproductive planning that better addresses perceptions towards fertility limitation. Nonetheless, results are consistent with findings from other investigations in West Africa distinguishing the region from reproductive preference and fertility regimes in Asia, and concluding that fertility transitions will be gradual, dependent upon spacing, and constrained by the absence of a widespread demand to limit family size [19].

Finally, it must be noted that while the questions used in this study are useful for predicting the course of fertility and guide reproductive health programs, they are often predicated on assumptions that decisions about fertility are solely based on women and in part, in conjunction with their partners’ aspirations. Notwithstanding the fact that to a very large extent these questions have served the desired purpose [11, 20, 21], it is important to recognize that they often tend to ignore extended familial and social pressures that impact on fertility decision making of women or couples, as well as broader societal values regarding childbearing.

## Conclusion

The results suggest that while progress may have been made in reducing mortality in both the Upper East region and the country, there has not been an equivalently concomitant improvement in fertility and reproductive outcomes. The persistently low levels of contraceptive use, high unmet need and high levels of fertility suggests that many of the reproductive health including family planning have not made much progressed as anticipated. The UER has been successful in scaling up community-based primary health care. Research on mortality effects of CHPS scale up indicates that childhood mortality has rapidly declined. Reasons for the persistence of low contraceptive use and pervasiveness of unmet need for contraception, despite the expansion of access to family planning services, merit careful investigation and concerted programmatic action in the future. In particular, research should focus on the possibility that CHPS activities are promoting awareness of family planning without providing convenient services or services that offset the social costs of reproductive planning.

## Abbreviations

CHPS: Community-based Health Planning and Services
GDHS: Ghana Demographic and Health Survey
GEHIP: Ghana Essential Health Intervention Program
TFR: Total Fertility Rate
UER: Upper East Region
